# Resection rectopexy as part of the multidisciplinary approach in the management of complex pelvic floor disorders

**DOI:** 10.1515/iss-2022-0027

**Published:** 2023-07-31

**Authors:** Georgi Kalev, Christoph Marquardt, Marten Schmerer, Anja Ulrich, Wolfgang Heyl, Thomas Schiedeck

**Affiliations:** Department of General, Visceral, Thoracic and Pediatric Surgery, Ludwigsburg Hospital, Ludwigsburg, Germany; Department of Obstetrics and Gynecology, Ludwigsburg Hospital, Ludwigsburg, Germany

**Keywords:** pelvic floor disorders, pelvic organ prolapse, rectal prolapse, rectopexy, resection rectopexy

## Abstract

**Objectives:**

Pelvic floor disorders are frequently caused by an organ prolapse involving multiple pelvic floor compartments. In such cases, a multidisciplinary strategy for diagnostic work-up and therapy is required.

**Methods:**

All patients who underwent transabdominal rectopexy/resection rectopexy alone or in combination with simultaneous gynecological pelvic floor reconstruction at our institution between 01/2006 and 12/2021 were included in this retrospective study. The study aimed to evaluate the functional outcome and postoperative complications.

**Results:**

Two hundred and eighty seven patients were assigned to one of the following groups: PG1 – patient group one: after resection rectopexy (n=141); PG2 – after ventral rectopexy (n=8); PG3 – after combined resection rectopexy and sacro (cervico)colpopexy (n=62); PG4 – after combined resection rectopexy and *trans*-vaginal pelvic floor repair (n=76). The duration of follow-up was 14 months for PG1 (median, IQR 37 months), 11 months for PG2 (mean, SD 9 months), 7 months for PG 3 (median, IQR 33 months), and 12 months for PG 4 (median, IQR 51 Months). The surgical procedure resulted in improvement of symptoms related to obstructed defecation in 56.4 % (22/39) of the patients in PG1, 25 % in PG2 (1/4), 62.5 % (20/32) in PG3, and 71.8 % (28/39) in PG4. “*De novo*” constipation was reported by 2.4 % (2/141) of patients from PG1. Improvement in fecal incontinence symptoms was reported by 69 % (40/58) of patients in PG1, 100 % in PG2 (2/2), 93.1 % (27/29) in PG3, and 87.2 % (34/39) in PG4. The recurrence rate for external rectal prolapse was 7.1 % in PG1, 50 % in PG2 (1/2), 2.7 % in PG3, and 6.3 % in PG4. A significant difference in terms of severe morbidity (grade ≥ IIIb) and mortality could not be determined between the non-interdisciplinary (PG1 with PG2) and interdisciplinary surgery (PG3 with PG4) (p=0.88, p=0.499).

**Conclusions:**

Based on our results, we can assume that combined surgery is as feasible as rectal surgery alone. In our study, combined interventions were effective and not associated with an increased risk of postoperative complications.

## Introduction

The prevalence of pelvic organ prolapse (POP) in women is reported to be up to 50 % [1]. Although genital prolapse is a common finding on gynecologic examination, only about 3–6 % of patients have symptoms [1, 2]. However, symptomatic patients often have a considerable degree of suffering and reduced quality of life. The prevalence of external rectal prolapse is reported to be approximately 0.5 %, with women and older patients being more commonly affected [3]. It is not uncommon for patients with rectal prolapse to have a complex pelvic floor disorder with additional prolapse of the genital organs. The treatment of these complex clinical pictures is demanding and requires a multidisciplinary approach for diagnostic work-up and surgical treatment.

## Materials and methods

Our single-institutional study was approved by our local Ethical Committee of the State Medical Chamber of Baden-Württemberg. All female patients aged 18 and over who underwent transabdominal rectopexy/resection rectopexy alone or in combination with a surgical procedure to correct a female genital prolapse in the Department of General and Visceral Surgery at the Ludwigsburg Hospital from January 2006 to September 2021 were retrospectively included in this study. The operations were performed by experienced senior physicians from our institution. A senior gynecologist specialized in pelvic floor surgery participated in interdisciplinary surgical interventions. The surgical techniques used are outlined below.

### Surgical procedures

#### Resection rectopexy

We perform a complete anterior (rectovaginal fascia) and posterior (presacral space) dissection of the mesorectum down to the pelvic floor until the upper edge of the external sphincter is reached, leaving the lateral ligaments completely intact. After sigmoid/rectal resection, the Douglas pouch is reconstructed by suturing the peritoneum to the left and right of the rectum. In this final step, a suture rectopexy is performed by attaching the anterior wall of the rectum to the peritoneum.

#### Ventral rectopexy

In this procedure, only an anterior dissection along the rectovaginal fascia is performed until the upper edge of the external sphincter is reached. A mesh is then placed and attached to the anterior wall of the rectum and then fixed to the anterior ligament over S1–S2. The peritoneum is then closed with a continuous suture to cover the mesh.

#### Combined resection rectopexy and sacro (cervico)colpopexy

Like above at first, a complete anterior and posterior dissection is performed. Then a sigmoid/rectal resection is performed transecting the rectum in the upper third. A Hysterectomy is carried out only in case of additional uterine pathology not related to POP. The posterior vaginal (cervical) wall is then anchored to the anterior ligament at the S1–S2 level using a Y-shaped mesh. During the fixation of the mesh to the anterior ligament, the vaginal vault is elevated to the correct anatomic position using a reusable EEA sizer placed inside the vagina. Finally, the peritoneum is closed and the rectopexy is carried out.

#### Combined resection rectopexy and trans-vaginal pelvic floor repair

This procedure begins with a resection rectopexy (anterior and posterior dissection described above). The next step is transvaginal reconstructive surgery for POP including anterior, posterior, or combined vaginoplasty with or without mesh. In some patients, a concomitant apical suspension procedure was performed.

### Clinical variables

The electronic medical records at our institution were queried for pre-, intra-, and postoperative data. The retrospective data collection included clinical parameters such as age, BMI, ASA, parity, past left-side hemicolectomy or rectal resection, previous surgical treatment for pelvic floor disorders, findings from MR defecography, length of hospital stay, and laboratory findings. From the perspective of colorectal surgeons, we evaluated symptoms related to defecation disorders or fecal incontinence. Fecal incontinence was defined as the inability to control bowel movements and was categorized according to the following widely used classification: Grade I – Inability to control flatus; Grade 2 – Inability to control liquid stool; Grade III – Inability to control solid stool [4]. For a diagnosis of obstructed defecation, at least two of the ROME IV diagnostic criteria for functional constipation had to be met: straining during defecation, lumpy or hard stools, the sensation of incomplete evacuation, the sensation of anorectal obstruction, manual evacuation, and fewer than three spontaneous defecations per week [5].

### Objectives and endpoints

The assessment of the symptoms related to obstructed defecation or fecal incontinence pre- and postoperatively was the first objective of this study and patient satisfaction after surgery was set as the first study endpoint. A three-point scale was used to evaluate the functional outcome – improvement/no change/worsening of the symptoms.

The second objective was to evaluate the success of the surgical reconstruction measured by the absence of recurrent rectal prolapse during the observation period in patients with preoperatively diagnosed external rectal prolapse. The second study endpoint was, respectively, the rate of recurrent rectal prolapse. First-or second-degree rectal prolapse preoperatively was not considered in the analysis.

This study also aimed to clarify whether interdisciplinary surgical management of complex pelvic floor pathology is associated with an increased risk compared with rectopexy or resection rectopexy alone, which we defined as the third endpoint of the study.

### Statistical analysis

The Kolmogorov-Smirnov and the Shapiro–Walk test were used to determine if the variables were normally distributed. For variables that were skewed and therefore not normally distributed, the Mann–Whitney U test was applied. For the analysis of categorical variables, we used the chi-square test, and for expected frequency less than five – the Fisher’s exact test. We chose a significance level α=0.05.

## Results

From January 2006 to September 2021 a total of 288 female Patients were operated on at our institution using one of the above-mentioned techniques. One patient with a pelvic floor defect due to a severe car accident was excluded. 287 patients were included in this study and assigned to one of the following groups: patient group one (PG1) – Patients after resection rectopexy (n=141); patient group two (PG2) – patients after ventral rectopexy (n=8); patient group three (PG3) – patients after combined resection rectopexy and sacro (cervico)colpopexy (n=62); patient group four (PG4) – patients after combined resection rectopexy and trans-vaginal pelvic floor repair (n=76). In the last group of patients, the following procedures were performed in addition to resection rectopexy: anterior vaginoplasty (AVP) in 11 patients with mesh reinforcement in 3 cases, posterior vaginoplasty (PVP) in 33 patients with mesh reinforcement in 23 cases, concomitant anterior and posterior vaginoplasty (AaPVP) in 30 patients with mesh reinforcement in 18 cases (in 2 cases anterior mesh, in 4 cases posterior mesh and in 12 cases combined). Some women in this group received an apical suspension procedure. One patient with AVP, 5 patients with PVP, and 5 patients with AaPVP underwent simultaneous vaginal sacrospinal fixation (according to Amreich-Richter). One patient with PVP underwent a bilateral sacrospinal colposuspension. TVT was performed in two patients from PG 4 after resection rectopexy.

The demographic and clinical characteristics of the patients are summarized in Table 1. There was no significant difference in the demographic characteristics (age, p=0.21; BMI, p=0.15; severe ASA score – III/IV, p=0.26) between the patients who underwent interdisciplinary and non-interdisciplinary surgery. Significantly more patients in the group after interdisciplinary surgery had a previous hysterectomy (p=0,000,001).

**Table 1:** Patient demographic and clinical characteristics, past history, surgical approach, and symptoms at presentation.

Variable	Non-interdisciplinary surgery			Interdisciplinary surgery	
	PG 1^a^		PG 2^a^	PG 3^a^	PG 4^a^
Number of patients	141		8	62	76
Age, years	67 (IQR 20)		64.1 (SD 13.4)	61.9 (SD 13.3)	66.4 (10.7)
BMI^a^, kg/m^2^	23 (IQR 6)		25.4 (SD 3.1)	24.2 (SD 3.6)	25 (IQR 5.3)
ASA^a^ score [no./%]				
ASA I ASA II ASA III ASA IV	13 (9.2 %) 96 (68.1 %) 32 (22.7 %) 0		1 (12.5 %) 4 (50 %) 3 (37.5 %) 0	3 (4.8 %) 48 (77.4 %) 11 (17.7 %) 0	3 (3.9 %) 59 (77.6 %) 13 (17.1 %) 1 (1.3 %)
Anal sphincter defect (on ultrasound) [no./%]	16 (11.3 %)		–	7 (11.3 %)	5 (6.6 %)
Vaginal births, % – 0 births – 1 birth – 2 births – 3 births – >3 births	14.5 % 19.1 % 37.3 % 19.1 % 10 %		55.6 % 11.1 % 33.3 % – –	17 % 23.4 % 34 % 19.1 % 6.4 %	10 % 20 % 34.3 % 17.1 % 18.6 %
Past hysterectomy	46 (32.6 %)		2 (25 %)	33 (53.2 %)	52 (68.4 %)
Concomitant hysterectomy	1 (0.7 %)		0	18 (29 %)	5 (6.6 %)
Past resection rectopexy [no./%]	3 (2.1 %)		1 (12.5 %)	3 (4.8 %)	1 (1.3 %)
Past ventral rectopexy [no./%]	0		0	2 (3.2 %)	0
Past transanal-perineal procedure [no./%] (Altemeier^b^, Rehn-Delorme^c^, STARR^d^)	9 (6.4 %)		0	3 (4.8 %)	2 (2.6 %)
Past abdominal procedure for POP	1^e^ (0.7 %)		0	0	4^f^(5.3 %)
Past transvaginal procedure for POP^e^	16 (11.3 %)		2 (25 %)	17 (27.4 %)	22 (28.9 %)
Highest postoperative level of CRPa, mg/L	44.7 (IQR 42.8)		16.5 (IQR 17.5)	44.9 (IQR 52.9	48.8 (IQR 42.8)

**Surgical approach**					

Laparoscopy [no./%]	113 (80.1 %)	7 (87.5 %)		31 (50 %)	70 (92.1 %)
Robotic-assisted [no./%]	14 (9.9 %)	1 (12.5 %)		25 (40.3 %)	1 (1.3 %)
Laparotomy [no./%]	1 (0.7 %)	0		3 (4.8 %)	2 (2.6 %)
Conversion to laparotomy [no./%]	13 (9.2 %)	0		3 (4.8 %)	3 (3.9 %)

**Leading preoperative symptoms**					

External rectal prolapse [no./%]	104 (73.8 %)		2 (25 %)	37 (59.7 %)	32 (42.1 %)
Fecal incontinence [no./%] – Grade I (flatus) [no.] – Grade II (liquid stool) [no.] – Grade III (solid stool) [no.]	93 (66 %) 38 35 20		3 (37.5 %) 1 1 1	39 (62.9 %) 9 12 18	48 (63.2 %) 17 14 17
Obstructed defecation [no./%]	57 (40.4 %)		4 (50 %)	36 (58.1 %)	50 (65.8 %)
Feeling a bulge from the anus [no./%]	9 (6.4 %)		–	4 (6.5 %)	1 (1.3 %)
Rectal mucus discharge [no./%]	15 (10.6 %)		2 (25 %)	7 (11.3 %)	2 (2.6 %)
Rectal bleeding [no./%]	12 (8.5 %)		2 (25 %)	6 (9.7 %)	5 (6.6 %)
Sensation of bulging in the vagina [no./%]	–		–	–	5 (6.6 %)

^a^PG 1, patient group one-patients after resection rectopexy; PG 2, patient group two – patients after ventral rectopexy; PG 3, patient group three – patients after combined resection rectopexy and sacro (cervico)colpopexy; PG 4, patient group four – patients after combined resection rectopexy and trans-vaginal pelvic floor repair; ASA, American Society of Anesthesiology physical status; BMI, body mass index; CRP, C-reactive protein; SD, standard deviation; IQR, interquartile range. ^b^Transanal-perineal rectal resection (full thickness resection). ^c^Transanal mucosal resection. ^d^Stapled transanal rectal resection (STARR). ^e^Vaginoplasty, colposuspension. ^f^Sacrocolpopexy. ^g^2× Sacrocolpopexy und 2× Burch Colposuspension. Values are given as mean (standard deviation), median (interquartile range), or absolute number of patients (percentage).

After discharge from the hospital, 60 % of the patients from PG1, 88 % from PG2, 84 % from PG3, and 80 % from PG4 presented for follow-up examinations, with a median follow-up of 14 Months for PG1 (IQR 37 Months), mean follow-up of 11 Months for PG2 (SD 9 Months), median follow-up of 7 Months for PG 3 (IQR 33 Months) and a median follow-up of 12 Months for PG 4 (IQR 51 Months). In patients with complex pelvic floor defects, outpatient follow-up appointments have been made more frequently in the gynecological or coloproctological outpatient clinic. This explains why more patients appeared for follow-up examinations in the multidisciplinary groups 3 and 4.

The average values of the measurements based on the MRI during the defecation phase are shown in Table 2.

**Table 2:** Preoperative findings on MRI.

	PG1	PG2	PG3	PG4		
				AVP^a^	PVP^a^	AaPVP^a^
n=^b^	76	7	58	10	26	22
Anterior compartment (cystocele), cm	1.9 (IQR 2)	2.1 (SD 1.5)	3.2 (SD 1.6)	4.6 (SD 1.2)	1.7 (SD 1.2)	3.3 (SD 1.3)
Middle compartiment (uterine/vaginal fault prolapse), cm	0.7 (IQR 1.9)	1.7 (SD 0.8)	3.1 (SD 1.5)	2.8 (SD 1.1)	1.6 (IGR 2.3)	2.3 (SD 1.6)
Posterior compartment (ARJ^c^ descent), cm	6.4 (IQR 1.7)	6.5 (SD 0.5)	7.4 (IQR 1.7)	6.8 (SD 1)	6.7 (SD 0.9)	6.8 (SD 0.9)
Rectocele, cm	1.7 (IQR 2.9)	1.9 (SD 2.4)	2.5 (IQR 3.7)	1.2 (SD 1.1)	3.4 (IQR 1.1)	3 (IQR 2)
Enterocele [no./%]	20 (26.3 %)	6 (85.7 %)	48 (82.8 %)	6 (60 %)	15 (57.7 %)	12 (54.5 %)

^a^AVP, anterior vaginal plastic; PVP, posterior vaginal plastic; AaPVP, anterior and posterior vaginal plastic. ^b^Number of patients who received an MRI preoperatively. ^c^Anorectal junctio. The pubococcygeal line (PCL), which extends in the sagital plane from the inferior border of the symphysis pubis to the last coccygeal joint, represents the approximate height of the pelvic floor and is considered the reference line for estimating POP [6, 7]. Anatomical reference points are defined in each pelvic floor compartment, in the anterior compartment – the most inferior border of the bladder base, in the middle – the most inferior edge of the cervix (the upper end of the vaginal stump in patients with previous hysterectomy), in the posterior – the anorectal junction [6, 7]. The average descent of the anterior, middle, and posterior compartments during the defecation phase is measured in the sagital plane as a perpendicular distance from the corresponding reference point in each compartment to the pubococcygeal line (PCL) [7]. The distance between the line that is drawn through the anterior wall of the anal canal and extended upwards, and the anterior rectal wall determines the size of the rectocele [7]. Enterocele is defined as herniation of the peritoneum into the rectogenital pouch containing fat, loops of small bowel, or the sigmoid colon [8]. Only by MRI confirmed enterocele diagnoses are shown in the table. Values are given as mean (standard deviation), median (interquartile range), or absolute number of patients (percentage)

The surgical procedure resulted in improvement of symptoms related to obstructed defecation in 56.4 % (22/39) of the patients in PG1, 25 % of the patients in PG2 (1/4), 62.5 % (20/32) in PG3, and 71.8 % (28/39) of the patients in PG4 (80 % in AVP (4/5); 53.3 % (8/15) in PVP and 83.3 % (15/18) in AaPVP, in one patient after TVT). “*De novo*” constipation occurred in 2 patients (2.4 %) in PG1 and no patients in PG2, PG3, and PG4. A reduction in incontinence episodes and improvement in fecal incontinence symptoms was reported by 69 % (40/58) of patients in PG1, 100 % of patients in PG2 (2/2), 93.1 % (27/29) of patients in PG3 and 87.2 % (34/39) of patients in PG4 (100 % in AVP (9/9); 81.3 % (13/16) in PVP and 84.6 % (11/13) in AaPVP, in one patient after TVT.

The recurrence rate after surgery for third-degree rectal prolapse was 7.1 % it the PG1. One patient from this Group underwent a Rehn-Delorme mucosectomy 7 years after resection rectopexy due to a mucosal prolapse. The recurrence rate for rectal prolapse was 50 % in PG2 (1/2), 2.7 % in PG3, and 6.3 % in PG4.

In PG3, 3 patients (4.8 %) underwent a renewed apical suspension procedure because of a recurrent vaginal stump prolapse. In another 3 patients (4.8 %) from this group, TVT was performed due to stress urinary incontinence.

In PG4, 2 patients (2.6 %) underwent repeat transvaginal surgery in the same compartment.

Postoperative surgical complications during hospitalization were assessed according to the Clavien-Dindo classification [9]. Patient groups were compared according to the number of severe complications encountered (Table 3). Complications classified as grade≥IIIb were considered severe: IIIb – complications requiring surgical, endoscopic, or radiological intervention under general anesthesia, IV – life-threatening complications with single organ dysfunction (IVa) or multiorgan dysfunction (IVb) requiring intensive care management, V – the death of a patient. Anastomotic leaks were categorized according to the clinical classification proposed by the International Study Group of Rectal Cancer, with those anastomotic leaks requiring re-laparotomy being defined as grade C [10].

**Table 3:** Postoperative complications according to the Clavien–Dindo classification.

Patient group	non-interdisciplinary surgery		interdisciplinary surgery	
	PG 1 (n=141)	PG 2 (n=8)	PG 3 (n=62)	PG4 (n=76)
Grade I	1 (0.7 %)	0	0	1 (1.3 %)
Grade II	0	0	0	0
Grade IIIa	1 (0.7 %)	0	2 (3.2 %)	0
Grade IIIb	4 (2.8 %)	1 (12.5 %)	2 (3.2 %)	6 (7.9 %)
Grade IVa	0	0	0	0
Grade IVb	1 (0.7 %)	0	0	0
Grade V	2 (1.4 %)	0	0	0
Total	9 (6.4 %)	1 (12.5 %)	4 (6.5 %)	7 (9.2 %)
	10 (6.7 %)		11 (8 %)	

Each cell contains the number of patients and in brackets the complication rate.

Severe postoperative complications occurred in 7 patients in PG 1: in two patients grade C anastomotic leakage occurred (one was classified as a grade IVb complication and one as a grade V complication according to the Clavien–Dindo classification), three patients underwent a surgical revision for intestinal obstruction due to an incarcerated trocar-site hernia (two were classified as grade IIIb complications and one as grade V complication) and two patients have been re-operated due to postoperative bleeding (both classified as grade IIIb complications).

In PG 2, one patient underwent relaparoscopy on the seventh day after surgery due to intestinal obstruction secondary to postoperative adhesion (grade IIIb complication).

During the inpatient stay, two patients from PG 3 underwent re-operation - one patient for an anastomotic leak (grade IIIb complication) and another for abdominal wound dehiscence after laparotomy (grade IIIb complication). In another patient in this group, the left ureter was accidently transected intraoperatively (intraoperative adverse event). The injury was recognized immediately and a prompt urologic consultation resulted in a robotic-assisted ureteroneocystostomy. This patient was discharged without further problems.

In PG4, three grade C anastomotic leaks occurred (each of these was classified as a grade IIIb complication), postoperative bleeding occurred in two patients (both classified as grade IIIb complications) and one patient underwent a relaparoscopy to evacuate an infected hematoma (complication grade IIIb).

A significant difference in terms of severe morbidity (grade≥IIIb) and mortality could not be determined between the non-interdisciplinary (PG1 with PG2) and interdisciplinary interventions (PG3 with PG4). (p=0.88 resp. p=0.499).

Mesh erosion is a specific late complication after mesh implantation. Three patients presented with mesh erosion of the vaginal wall at an average of 26 months after sacrocolpopexy (PG3). Transvaginal partial mesh resection with the closure of the vaginal wall defect was successful in 2 patients. Laparoscopic partial mesh resection was performed in one patient.

Mesh erosion occurred in three patients in the PG4 group, on average 27 months after mesh application. Two of the patients underwent transvaginal partial mesh resection and a combined transvaginal and transvesicular resection was performed in one patient.

## Discussion

The complete (full-thickness) external rectal prolapse is defined as the protrusion of the entire rectal wall through the anal canal. Occult (internal) rectal prolapse or rectal intussusception is when the rectal wall is prolapsed but does not protrude through the anus. An isolated mucosal prolapse must be differentiated from a full-thickness rectal prolapse [11]. Traditionally, POP is considered a disorder that affects the female genital organs. It is defined as a protrusion of the uterus, vaginal apex, bladder, rectum, or intestines into the vagina causing a bulging of the vaginal walls [12]. To obtain a better overview the female pelvic floor is divided into three compartments: the anterior compartment (bladder and urethra), the middle (apical) compartment (uterus, vagina), and the posterior compartment (rectum) [13]. The pelvic floor with its complex anatomy forms a functional unit together with all pelvic organs. Folkmann found in his patient cohort that 21 % of the patients with urinary incontinence, pelvic organ prolapse, or both had fecal incontinence to a degree that was considered a problem by the patients [14]. Jelovsek reported a higher prevalence of functional anorectal disorders in patients with advanced pelvic organ prolapse (POP) [15]. In another study, a higher prevalence rate of urinary incontinence (58 %) and pelvic genital prolapse (24 %) was found in females who underwent surgery for either fecal incontinence or rectal prolapse compared with the control group [16]. Therefore, functional anorectal disorders, rectal prolapse, and POP should be considered closely related entities. The coexistence of these pathologies, which is not uncommon, determines the need for a multidisciplinary approach to diagnosis and therapy.

The clinical examination alone, especially in patients with moderate to severe symptoms, frequently underestimates the number of the involved compartments [6]. This fact underlines the important role of MRI defecography as a dynamic imaging modality to evaluate the morphology as well as the functional disorders of the pelvic floor. This special MRI examination is divided into the following phases – rest, squeeze, strain, and defecation phase [6]. On one hand, the perpendicular distance from the reference point of each pelvic floor compartment to the PCL is measured in all phases; on the other hand, the dynamic change in the position of the pelvic organs during defecation is evaluated [6]. During defecation, the pelvic floor is under maximum stress – the highest intra-abdominal pressure is reached while the levator ani muscle is maximally relaxed. This potentiates the detection of a POP and explains the high sensitivity of the defecation phase [6, 17].

Various surgical procedures for the treatment of external rectal prolapse have been described in the literature, generally divided into two groups – procedures with perineal or transabdominal approach [18]. Although the evidence is limited, most authors favored perineal access in elderly and multimorbid patients because of less perioperative morbidity, less pain, and a shorter hospital stay. However, the recurrence rate is reported to be 4-fold higher than for transabdominal procedures in various reports [18]. Therefore, transabdominal techniques are recommended for young and healthy patients [18]. Laparoscopy with or without robotic assistance reduces the disadvantages of open surgery. Due to the growing use of minimally invasive procedures, transabdominal techniques are also increasingly used in older patients and represent the vast majority of surgical interventions at our institution. The mobilization of the rectum is an important step that can be performed ventrally, dorsally, or in combination. To date, it is unclear which procedure is superior. In ventral mesh rectopexy (D’Hoore procedure) the dissection is limited to the anterior aspect of the rectum and no posterolateral mobilization is performed [19]. According to the authors, postoperative constipation was prevented in their patient population by avoiding complete rectal mobilization, which might be associated with an increased risk of autonomic nerve damage [19]. We also advocate the preservation of the lateral ligaments, which is reported to be associated with less postoperative constipation [20]. In our opinion, the combined anterior and posterior rectal mobilization allows full straightening of the rectum. The additional resection of the sigmoid colon leads to the improvement of constipation symptoms and prevents the occurrence of new ones [18]. Resection rectopexy alone without mesh implantation provides less stabilization of the middle pelvic floor compartment compared to ventral rectopexy. Therefore, we perform resection rectopexy in combination with sacro (cervico)colpopexy in patients with additional prolapse in the middle compartment.

In recent years, multidisciplinary therapy for simultaneous genital and rectal prolapse has become increasingly popular. Dekel et al. included 10 patients with concurrent genital and rectal prolapse in their study and report that a joint transperineal operation – Joel-Cohen vaginal hysterectomy followed by the Altemeier procedure – is safely feasible and efficient in terms of recurrence and continence [21]. In another paper with a total of 142 patients included, the clinical and functional outcomes of STARR were compared with those of STARR plus concomitant pelvic floor surgery – transobturator tape (TVT-O), vaginal repair of enterocele, or vaginal hysterectomy [22]. The authors noted no recurrences 2 years after surgery and considered the combined surgery to be as effective as rectal surgery alone. The overall patient satisfaction index at 2 years showed no significant differences between the two groups. Nevertheless, higher morbidity was reported in the vaginal hysterectomy group. In a study from Australia, eighty-nine patients underwent combined surgery for rectal prolapse and post-hysterectomy vaginal vault prolapse [23]. The operative procedure consisted of an abdominal mesh rectopexy of the Wells or Ripstein II type, abdominal closure of the pelvic cul-de-sac with a series of abdominally placed concentric Moschcowitz pursestring sutures, and a colpopexy. All major symptoms improved, especially the pelvic pain and there was no recurrence of rectal or vaginal vault prolapse. In a retrospective review, Watadani et al. evaluated the outcomes of sacrocolpopexy and rectopexy by comparing preoperative and postoperative function and quality of life [24]. 110 women underwent sacrocolpopexy and rectopexy and 33 concomitant hysterectomies. 96 patients had a rectal prolapse and 14 had a rectal intussusception. Each patient had also either enterocele (n=86) or vaginal prolapse (n=48). The following complications occurred – presacral bleeding (n=2), ureteral injury (n=2), wound infection (n=8), and pulmonary embolism (n=2). No mortalities occurred. Postoperatively, 82 % reported improvement or resolution of symptoms. Quality-of-life scores improved significantly. No recurrent rectal prolapse occurred [24]. In 3 further studies, the combined rectopexy and sacrocolpopexy for multicompartment pelvic organ prolapse was investigated. Campagna et al. observed significant improvement in symptoms related to POP and obstructed defecation syndrome and reported no intraoperative or postoperative morbidity in their patient cohort of 98 patients [25]. A smaller study with 29 patients after open mesh sacrocolporectopexy confers good symptomatic improvement for urinary-, vaginal-, and rectal-related symptoms. One patient developed a serious complication from mesh erosion into the vagina [26]. A study from 2018 focused on the safety of the combined intervention. The authors found no significant difference in operative morbidity when sacrocolpopexy is added to the rectopexy procedure (14.8 % rectopexy alone vs. 13.6 % combined procedure) [27].

In our study, the patient groups after interdisciplinary and non-interdisciplinary surgical interventions were homogeneous concerning the demographic characteristics (age, BMI, ASA score). As expected, significantly more patients in the group which received an interdisciplinary surgery had undergone a hysterectomy in the past. The latter is associated with an increased risk for pelvic genital prolapse, which was also confirmed by our data.

At our institution, dynamic MRI is the standard diagnostic for the evaluation of any complex pelvic floor pathology and is crucial for the selection of surgical therapy. If there is evidence of an external rectal prolapse with a concomitant prolapse in the middle and/or the anterior compartment, we perform a combined surgical intervention. Following the reports from the last years, our data also show the high effectiveness of interdisciplinary surgery for complex pelvic floor disorders with or without a concomitant external rectal prolapse. The majority of the patients in our study reported an improvement in their symptoms related to obstructed defecation or fecal incontinence. In the literature, the recurrence rate of rectal prolapse is given as 10 %–20 %. The recurrence rate after surgery for recurrent prolapse is even higher, at 29 % [24]. The low recurrence rate of external rectal prolapse in our patient population, which was our second study objective, also indicates the high effectiveness of surgical procedures. In addition, the rare need for reoperation for POP in the same pelvic floor compartment may indirectly indicate successful surgery.

We found no significant difference in the rate of postoperative complications between the interdisciplinary procedures and the (resection) rectopexy alone in our patient population. Overall, the complication rate was low in all patient groups. Three of the severe complications in PG1 were due to incarcerated herniation of the small bowel after the laparoscopic procedure which had always occurred at the site of the 13 mm trocar in the right lower abdomen. Previously, a drain had been routinely placed at the site of this trocar, and the opening in the fascia had not been closed. After the last adverse event, the fascia was always closed and this complication has not recurred since 2015. Mesh erosion has rarely occurred in our patients. This late complication was treated in most cases by transvaginal partial mesh resection. A re-laparoscopy was required in only one case.

This study has some limitations. First, it was a retrospective study conducted at a single center. Second, we did not use elaborated and established scores on fecal incontinence or obstructed defecation. We can report very good results based on our data, but these are not directly comparable to the results of other studies. Third, we did not perform postoperative control dynamic MRI defecography in the majority of patients to confirm the restoration of the anatomic integrity of the pelvic floor. In our opinion, however, this is of minor importance because the improvement in the patient’s symptoms is the most important indicator of the success of the procedure.

Our data demonstrate that pelvic floor surgery can be successfully and safely performed minimally invasively with the increasing use of robotic surgical systems in recent years. After the introduction of the robotic system in our hospital (June 2016), a large part of the procedures were performed robotically assisted – 67 % in PG1, 100 % in PG2 (one patient since June 2016), 83 % in PG3, and 100 % in PG4 (one patient since June 2016). It is interesting to note that out of a total of 41 robotically assisted procedures, we have not had a single conversion, while laparoscopic procedures had a conversion rate of 10 % in PG1, 0 % in PG2, 9 % in PG3, and 4 % in PG4. Whether the use of robotic systems will provide an advantage in terms of functional outcomes remains to be seen in the near future.

## Supplementary Material

Supplementary Material
