# Supplementary material for: Resection rectopexy as part of the multidisciplinary approach in the management of complex pelvic floor disorders
Source: Innov Surg Sci. 2023 Jul 31;8(1):29–36. doi: 10.1515/iss-2022-0027 (PMC10576551; doi:10.1515/iss-2022-0027)
Supplement: Supplementary file 1 — Supplementary Material [file j_iss-2022-0027_suppl_001.pdf]

Reviewer Assessment

G. Kalev et al.: Resection rectopexy as part of the multidisciplinary approach in the management of complex pelvic floor disorders

Reviewers' Comments to Original Submission

Reviewer 1: anonymous

Date received: 02-Apr-2023
Reviewer recommendation: **Accept in present form**
Reviewer overall scoring: **High**

Comments to author:
Very nice and interesting publication of a great experience. Very worth publishing in an open access journal..

Reviewer 2: Kneist, Werner

Date received: 22-Apr-2023
Reviewer recommendation: **Return to author for major modifications**
Reviewer overall scoring: **High**

Comments to author:
This interesting retrospective monocentric study aims to assess value of resection rectopexy in patients with symptomatic pelvic floor dysfunction. The main objective was to prove the patient satisfaction after surgery in a designated pelvic floor center.
For this purpose the authors compared 287 consecutive resection- mesh or resection- rectopexies that were operated by experienced colorectal surgeons within 15 years. Patients were assigned to one of four groups. 138 (48%!) patients underwent interdisciplinary surgery simultanously.
Follow-up was in-clinic and timing varied in groups between median 7 and 14 months with large interquartile ranges. Group-specific, the rate for missed follow-up examinations was 12-40 percent. The follow-up patient-reported outcome measure data are presented by pragmatic clinical grades, based on the ROM criteria and as 3-point symptom scale values (worse, same, better).

In addition, MRI examination data before surgery and rate of recurrent external rectal prolapse and complication rates postoperatively was carried out. Reference is made to the techniques of resection-rectopexy, ventral-mesh-rectopexy and concomitant gynecological operations are therefore comprehensible.

The consideration of pelvic floor dysorders und and their surgical treatment corresponds to clinical reality and, especially for the entity of rectal prolapse, can be compared well with the literature. The authors are to be congratulated on their consistent scientific examination of a complex and interdisciplinary surgical field.
They initially conclude that combined (interdisciplinary) surgery is not associated with a higher risk in comparison to colorectal surgery alone. Furthermore, very good results are also reported for recurrence rate of external prolaps.

Preoperative MRI examinations of the extensive and well-characterized series form a good comparison for other working groups interested in pelvic surgery and should therefore remain included. The same applies to the main results of female patient related outcomes.

It is certainly a very interesting retrospective study with presentation of very interesting perioperative data and consistently complemented by the thorough evaluation of the mid-term functional follow-up. This is very important as prospectively recorded functional data (especially for patients with simultaneous interdisciplinary operations) are rare. Against this background, well-processed retrospective data also have their value. They are important for assessing the quality of life of our pelvic floor patients.
However, questions and concerns still arise that should be considered by the authors.

Major points
The treatment groups appear well balanced with respect to baseline demographic and clinical characteristics. However, the selection of patients for the procedure of resection rectopexy vs mesh rectopexy according to their disorder requires more explanation. What “advantages and disadvantages” of the procedures were discussed?

What explains the differences in follow up 60% percent (group 1) vs > 80% (group 2-4). Does pelvic floor centralization and interdisciplinary follow-up play a role? Are there other explanations (e.g. age, complication, etc)?

Since the authors emphasize the value of robotics also with regard to the increasing use, this should also be addressed more directly in the discussion. Thinking about robotics within an interdisciplinary innovative approach, the numbers should also be given over study period. (effect?).

The discussion part and the introductory part must be shortened.

Minor points

• However, you could possibly exclude the patients with mesh rectopexy (group 2) to streamline the paper. The title and the low number of cases (n= 8) justify addressing this point briefly in the discussion in order not to lose the overall picture of your colorectal spectrum.

• Page 4 line 58: patients instead of Patients

• Page 7 line 60: Watadani et al. instead of Watadani

Authors’ Response to Reviewer Comments

Date received: 25-Apr-2023

First of all, I would like to thank you for your helpful comments.

In the following, I will reply point-by-point to these comments:

Major points

1. The treatment groups appear well balanced with respect to baseline demographic and clinical characteristics. However, the selection of patients for the procedure of resection rectopexy vs mesh rectopexy according to their disorder requires more explanation. What “advantages and disadvantages” of the procedures were discussed?

Reply:

In part, we discussed this issue on page 7, lines 24-34. We believe that both anterior and posterior mobilization of the rectum during resection rectopexy allows for full extension of the bowel, which is essential for resolving constipation symptoms. Therefore, we consider this to be an advantage of resection rectopexy over ventral rectopexy, and it is preferred by us in patients with a stable middle compartment.

On the other hand, ventral rectopexy provides additional stabilization of the middle pelvic floor compartment compared with resection rectopexy alone without mesh implantation. In patients with additional prolapse of the middle compartment, we advocate resection rectopexy combined with sacrocolpopexy. In our opinion, fixation of the mesh to the posterior wall of the vagina or to the cervix in sacro(cervico)colpopexy brings a lower risk of infection compared with mesh fixation to the anterior rectal wall in ventral rectopexy.

Corresponding Text was added to the discussion.

2. What explains the differences in follow up 60% percent (group 1) vs > 80% (group 2-4). Does pelvic floor centralization and interdisciplinary follow-up play a role? Are there other explanations (e.g. age, complication, etc)?

Reply:

In patients with complex pelvic floor defects, outpatient follow-up appointments have been made more frequently in the gynecological or coloproctological outpatient clinic. In addition, these patients also have more often micturition disorders that required follow-up examinations. The last ones are not among the objectives of this study. This explains why more patients appeared for a follow-up examination in the multidisciplinary groups 2-4.

Corresponding Text was added to the results.

3. Since the authors emphasize the value of robotics also with regard to the increasing use, this should also be addressed more directly in the discussion. Thinking about robotics within an interdisciplinary innovative approach, the numbers should also be given over study period. (effect?).

Reply:

The number of robotic-assisted procedures in each of the studied groups is listed in Table 1. Indeed, it is interesting to note the proportion of these procedures after the introduction of the robotic system in our hospital and not for the entire study period. We do not expect a statistically relevant difference in functional outcomes because the procedure has not changed. What should be noted, however, is that there was a 0 percent conversion rate in a total of 41 robot-assisted interventions.

Corresponding Text was added to the discussion.

4. The discussion part and the introductory part must be shortened.

Reply:

The discussion part and the introductory part have been shortened.

Minor points

1. However, you could possibly exclude the patients with mesh rectopexy (group 2) to streamline the paper. The title and the low number of cases (n= 8) justify addressing this point briefly in the discussion in order not to lose the overall picture of your colorectal spectrum.

Reply:

The group of patients with ventral rectopexy is small, but we would like to keep it. On the one hand, this procedure is used in some centers as a standard procedure and therefore this surgical technique should be represented as one of the main groups. On the other hand, the eight patients are included in the analysis of complication rates in coloproctological procedures.

2. Correction done

3. Correction done

Reviewers’ Comments to Revised Submission

Reviewer 1: anonymous

Date received: 09-May-2023
Reviewer recommendation: **Accept in present form**
Reviewer overall scoring: **High**

Comments to author:

none.

Reviewer 2: Kneist, Werner

Date received: 15-May-2023
Reviewer recommendation: **Accept in present form**
Reviewer overall scoring: **High**

Comments to author:

The Dear authors,

Congratulations on the interesting scientific review of the extensive collective of patients. Thank you for answering my questions in detail and including them in the R1 manuscript.

Kind regards
